# Breakthrough in porous liquids for carbon capture and catalysis

**DOI:** 10.1093/nsr/nwaf075

**Published:** 2025-02-28

**Authors:** Weijin Li, Soumya Mukherjee

**Affiliations:** MIIT Key Laboratory of Advanced Display Materials and Devices, Jiangsu Province Engineering Research Center of Quantum Dot Display, School of Materials Science and Engineering, Institute of Optoelectronics & Nanomaterials, Nanjing University of Science and Technology, China; Department of Chemical Sciences, Bernal Institute and Research Ireland Centre for Pharmaceuticals (SSPC), University of Limerick, Ireland

Carbon dioxide (CO_2_) emissions remain a pressing environmental challenge, underscoring the urgent need for innovative, energy-efficient solutions. In line with national and global commitments to climate action, advancing carbon capture and conversion technologies is mission-critical to sustainably reducing emissions and achieving a net-zero future. In this context, a recent study by Yuan-Biao Huang and co-workers has introduced a disruptive material—a prototypical covalent organic framework (COF)-based smart porous liquid (PL), **COF-301-PL**, that merges the benefits of solid COFs and liquid materials, enabling efficient CO_2_ capture and heterogeneous catalysis [1].

**Figure 1.**
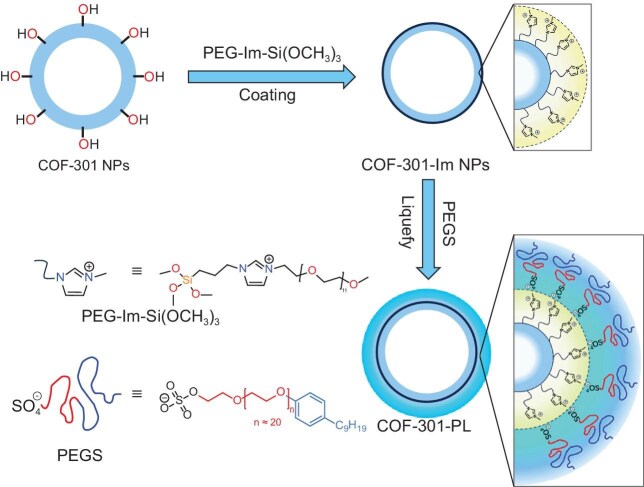
Schematic synthesis of the porous COF liquid **COF-301-PL**. NPs, nanoparticles.

**COF-301-PL** is prepared in two steps: (1) surface grafting of a three-dimensional microporous COF, **COF-301**, with positively charged polyethylene glycol (PEG) and an organosilane co-functionalized imidazolium salt (PEG-Im-Si(OCH₃)₃); and (2) liquefaction via anchoring of a negatively charged PEG-tailed sulfonate (PEGS) canopy through ion exchange (Fig. 1). This ensures that **COF-301-PL** retains high microporosity (for gases such as CO_2_) while maintaining its fluidity. What makes **COF-301-PL** exceptional is its liquid-like properties and ‘*breathing effect*’, meaning its excellent mass transfer capability in the liquid phase, alongside the dynamic expansion and contraction of its pores in response to CO_2_ pressure. This feature enhances CO_2_ storage, accelerates mass transfer, and optimizes catalytic efficiency, making it superior to traditional solid adsorbents under identical conditions.

Thanks to its strong CO_2_ capture ability, **COF-301-PL** serves as a gas reservoir for excellent CO_2_ conversion in the presence of the catalyst tetrabutylammonium bromide. It dramatically improves the transformation of CO_2_ and epoxide molecules into valuable cyclic carbonates, used in manufacturing plastics and pharmaceuticals. Compared to its solid COF counterpart and PEGS alone, **COF-301-PL** demonstrates 17-fold and 24-fold increases in catalytic efficiency, respectively. This advantage arises from **COF-301-PL**’s ability to store CO_2_ at high pressure and gradually release it, acting as a self-sustaining micro-reservoir for catalytic reactions. This eliminates the need for continuous CO_2_ supply, making industrial processes more efficient and energy-saving.

In essence, the development of **COF-301-PL** marks a milestone in porous liquid research. Its ability to reversibly adjust its pore structure to environmental conditions offers vast potential for improving industrial CO_2_ capture, chemical synthesis, and gas separation technologies. Future advances in this area could enable the porous liquids’ translation into large-scale commercial adoption. By combining structural flexibility with high adsorption and catalytic performance, **COF-301-PL** exemplifies how the disruptive material design of combining fluidity and porosity can profoundly impact the challenge of carbon capture and utilisation (CCU) [2].
